# Mimickers of pituitary tumors

**DOI:** 10.1093/noajnl/vdae085

**Published:** 2025-01-02

**Authors:** M Citlalli Perez-Guzman, Gabriela Szuman, Abdulrahman Altwijri, Kevin Shek, Sylvia L Asa, Shereen Ezzat

**Affiliations:** Endocrine Oncology Site Group, Princess Margaret Cancer Centre, Department of Medicine, University Health Network and University of Toronto, Toronto, Ontario, Canada; Endocrine Oncology Site Group, Princess Margaret Cancer Centre, Department of Medicine, University Health Network and University of Toronto, Toronto, Ontario, Canada; Endocrine Oncology Site Group, Princess Margaret Cancer Centre, Department of Medicine, University Health Network and University of Toronto, Toronto, Ontario, Canada; Joint Department of Medical Imaging, University Health Network and University of Toronto, Toronto, Ontario, Canada; Department of Pathology, University Hospitals Cleveland and Case Western Reserve University, Cleveland, Ohio, USA; Endocrine Oncology Site Group, Princess Margaret Cancer Centre, Department of Medicine, University Health Network and University of Toronto, Toronto, Ontario, Canada

**Keywords:** hypophysitis, immunotherapy, pituitary, pituitary neuroendocrine tumors, sellar lesion

## Abstract

Enlargement of the pituitary gland and/or its surrounding structures on brain or sellar imaging is a frequent finding. The distinction between clinically relevant and incidental changes can be challenging. Furthermore, the assumption that sellar lesions reflect a true neoplasm must be rigorously questioned to avoid inappropriate treatment or unnecessary surveillance plans. Here we review wide-ranging conditions that can mimic primary pituitary tumors. We outline a suggested approach to rational decision-making.

Imaging of the brain and skull base is commonly performed for a myriad of clinical indications. These can range from investigations for headaches to head trauma assessment. As with other body sites, radiographic changes in and around the sella turcica are typically reported to alert clinicians to abnormal imaging findings. Distinguishing clinical features from primary pituitary neoplasms need to be considered when encountering such lesions. Unlike pituitary tumors that typically evolve with clinical and radiographic footprints over years, lesions with a more acute or atypical timeline should represent a red flag. Additional features include loss of posterior pituitary function with diabetes insipidus as well as thickening of the pituitary stalk.

A number of neoplasms can arise in the sella. The most common are the tumors of neuroendocrine cells of the adenohypophysis formerly known as “adenomas” and now recognized as pituitary neuroendocrine tumors; another recognized tumor type, craniopharyngiomas, arise from Rathke’s cleft precursors of the adenohypophysis. Other tumors can arise from components of the hypothalamus and posterior lobe or neurohypophysis. In addition, there are primary tumors of the meninges, bone, fibrous and vascular tissues, smooth muscle and skeletal muscle, nerves, and paraganglia. It is important to recognize that primary hemato-lymphoid tumors can arise in this location, as can germ cell tumors; the pituitary is also frequently the site of metastatic disease. Various cysts can also occur in this region, the most common being Rathke’s cleft cyst.^1^ In this review, we will focus on non-neoplastic conditions that can give rise to enlargement of the pituitary on imaging with variable impact on the function of this gland. A summary of these conditions is provided in Table 1. The reader is referred to other excellent reviews on primary tumors of the pituitary^25^ and metastases to this gland.^26^

**Table 1.** Spectrum of Non-neoplastic Conditions Giving Rise to Pituitary Enlargement

Condition	Clinical features	Histomorphologic features	MRI findings	Serologic markers	Treatment
*I. Hyperplasia*					
Primary hypothyroidism^2,3^	Female > Male History of autoimmune disease No headache, no panhypopituitarism.	Pituitary hyperplasia thyrotroph and/or lactotroph cells.	Pituitary enlargement Symmetric	TSH Free T4	Levothyroxine
Primary adrenocortical insufficiency^4^	Female > Male History of autoimmune disease No headache, no panhypopituitarism.	Hyperplasia of the corticotroph cells.	Pituitary enlargement Symmetric	ACTH Cortisol 21-Hydroxylase antibodies	Hydrocortisone, Fludrocortisone
Puberty^5,6^	Female > Male No headache, no panhypopituitarism.	Hyperplasia of the mammsomatotroph cells.	Pituitary enlargement Symmetric	---	Continued surveillance
Pregnancy^7^	No headache, no panhypopituitarism.	Hyperplasia of the lactotroph cells	Pituitary enlargement Symmetric	Beta-HCG	Continued surveillance
*II. Inflammatory*					
Lymphocytic hypophysitis^8,9^	Female > Male Pregnancy Postpartum History of autoimmune disease Headache ± Vision Anterior Hypopituitarism Diabetes insipidus (uncommon)	Infiltration by lymphocytes, including reactive follicles, plasma cells, and variable amounts of fibrosis. Necrosis can be either nonspecific or specific for certain cell types of chromogranin or synaptophysin. CD45 stains.	Diffuse and symmetrical gland enlargement, thickened stalk Loss of posterior pituitary bright spot in T1-weighted images, and intense homogenous enhancement, with a symmetrical suprasellar extension	Pituitary hormones	Consider glucocorticoids If resistant: Glucocorticoids Resistant: azathioprine, methotrexate, cyclosporine A, and rituximab Surgery
Granulomatous hypophysitis^9^	Female > Male Primary (isolated) or secondary to Sarcoidosis, Wegner, LCH, Syphilis tuberculosis Anterior Hypopituitarism ± diabetes insipidus ++Headache	Histiocytes, multinucleated giant cells, and lymphoplasmacytic inflammation	Hypothalamic-pituitary or stalk thickening Usually, diffuse enlargement of the pituitary gland, in homogeneously hyperintense on T1-W postcontrast sequences.	ACE Quantiferon ANCAs Pituitary hormones	Glucocorticoids in primary (less glucocorticoids -responsive) Specific treatment according to the underlying granulomatous disease
IgG4 hypophysitis^10,11^	Male > Female Pituitary-isolated 4%–5% Multisystemic Retroperitoneal fibrosis Lachrymal and parotid infiltration Polyadenopathy Pancreatitis	Lymphoplasmacytic infiltrate with an increased number of plasma cells, predominantly of the IgG4 subtype, fibrosis, and obliterative phlebitis.	Enlargement of the pituitary, with optic chiasmal compression. Thickened pituitary stalk, or mass formation in the infundibulum. Disappearance of physiologic posterior pituitary bright spot on T1-MRI is also common	IgG4 levels Pituitary hormones	Glucocorticoids/azathioprine Decompressive surgery
Immunotherapy-related^12–15^	Male > Female +++ Asthenia Headaches Anterior Hypopituitarism (insolate ACTH deficiency) Diabetes insipidus is uncommon. Ipilimumab Nivolumab Pembrolizumab Telimomab	Diffuse infiltration with lymphocytes and macrophages	Pituitary enlargement (mild) homogeneous or heterogeneous and can be accompanied by stalk thickening	Pituitary hormones	Glucocorticoids May need to switch class of immune checkpoint inhibition/immunotherapy
Xanthomatous Hypophysitis^16–18^	Female > Male Secondary to ruptured Rathke’s cleft cysts	Diffuse infiltration by lipid-rich histiocytes	Pituitary enlargement with cystic components, and peripheral contrast enhancement is observed	Pituitary hormones	Glucocorticoids Less responsive to corticosteroid therapy
Abscess^19–21^	Male > Female ± Hx of skull base surgery Fever meningitis Headaches Oculomotor palsy Hypopituitarism ± Diabetes insipidus	Bacterial agent usually gram + Acute or a chronic inflammatory process without cellular debris.	Cystic or partially cystic pituitary mass with T1 signal hypointensity/isointensity, T2 signal isointensity/hyperintensity, and peripheral rim enhancement after gadolinium injection.	Pituitary hormones	Surgical abscess drainage + antibiotic treatment according to the result of cultures obtained during surgery.
SARS2/COVID 19^22,23^	Male = Female Pregnancy Preexisting tumor Pituitary apoplexy: ++ + Headache Vision abnormalities Diplopia	Pituitary widespread degenerative features (ie, necrosis and hemorrhage)	Preexisting lesion with a hyper-intense area (high T1-signal consistent with intralesional bleeding)	PCR SARS2/COVID-19 Pituitary hormones	If pituitary apoplexy: may need decompressive surgery TSS
*III. Infiltrative*					
Hemochromatosis^24^	Male = Female Hormonal deficiencies	Iron overload	The pituitary stalk can appear enlarged, low signal intensity of the anterior lobe on T1 and T2 with lack of normal contrast enhancement	Iron Ferritin Transferrin Pituitary hormones	Phlebotomy, chelation therapy, hormonal replacement.

## Hyperplasias

As with other endocrine organs, adaptive pressures can alter hormone production, driving an increase in cell number and size, leading to glandular enlargement. The pituitary is no exception.

### Physiologic Pituitary Enlargement

The pituitary is the subject of mammosomatotroph and lactotroph hyperplasia during pregnancy and puberty.^27^ The time required for such changes to resolve varies considerably.^28^ This can provide a diagnostic challenge especially when confounded by clinical concerns. An example would be an athlete in their 20s who presents with headaches and/or menstrual disturbances. Both endocrine assessments, as well as imaging features, can be difficult to discern from a true primary pituitary neoplasm. Cautious interpretation and close surveillance can provide the necessary evidence for the correct diagnosis.

### Pathological Hyperplasia

The pituitary develops hyperplasia in conditions where there is a loss of negative feedback such as primary hypothyroidism^29^ and primary adrenocortical insufficiency.^4^ These hyperplastic lesions can be mistaken for a primary pituitary tumor.^30^ Restoration of target hormone levels usually results in regression of the size of the gland over time.^31,32^

Pathological conditions have also been reported as a cause of pituitary hyperplasia, such as ectopic acromegaly and Cushing’s syndrome due to CRH. Acromegaly resulting from ectopic production of growth hormone (GH)-releasing hormone (GHRH) is an extremely rare condition. The excess of GHRH produces an enlargement of the pituitary gland due to somatotroph hyperplasia.^33^ Similarly, in patients with Cushing syndrome due to ectopic CRH production, the excess of ectopic CRH leads to pituitary corticotroph hyperplasia.^34^

## Inflammatory Conditions

Inflammation of the pituitary can be due to autoimmunity, infection, or other reactive causes. There are several morphologic types that are associated with different pathogenetic mechanisms.

### Lymphocytic Hypophysitis

#### Clinical setting.—

Being the most common form of primary hypophysitis, this is typically encountered in patients with altered immune function manifested as an autoimmune condition.^8^

#### Description and diagnosis.—

**Figure 1.**
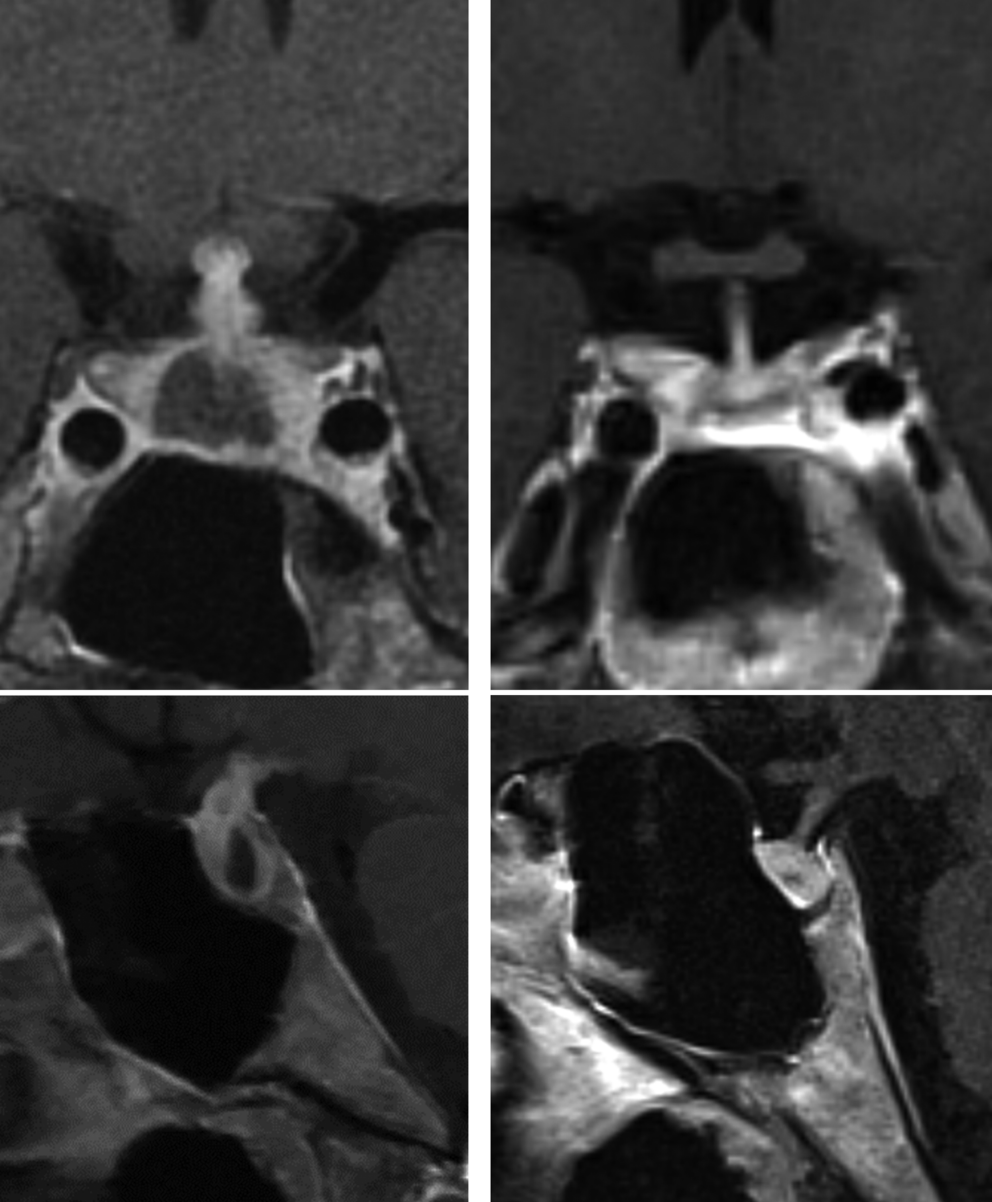
Lymphocytic hypophysitis—imaging. Postcontrast T1 pituitary magnetic resonance imaging coronal (top) and sagittal (bottom). Left, baseline. There is diffuse enlargement of the pituitary gland, with a central hypoenhancing area. There is also diffuse thickening and enhancement of the pituitary stalk and faint enhancement along the inferior margin of the hypothalamus. Right, 7 years after initial presentation, complete resolution of stalk thickening and gland enlargement.

This entity has been recognized for decades.^35^ It most typically presents in conjunction with altered immune status including the latter part of pregnancy.^35,36^ However, subsequent reports have described histologically-proven cases in other settings including older men^37^ and those with a wide spectrum of rheumatologic conditions.^38^ The clinical setting is probably the most instructive feature of this entity as the radiographic appearance of the pituitary can be extremely variable. This ranges from thickening of the pituitary stalk to transient global enlargement of the gland (Figure 1). Loss of the posterior bright spot, while initially considered to be diagnostic, has proven to be unreliable with poor diagnostic accuracy.^39^ Furthermore, the correlation between loss of this signal and functional clinical consequences such as diabetes insipidus is far from strict. A few reports of Infundibulohypophysitis have been documented; importantly, the presence of diabetes insipidus has been associated with a poor prognostic factor of response to glucocorticoids.^40,41^

The detection of various pituitary antibodies has been widely examined for diagnostic purposes.^42,43^ To date, however, the performance characteristics and predictive value of such serologic studies remain to be proven.^44,45^

#### Therapeutic management.—

**Figure 2.**
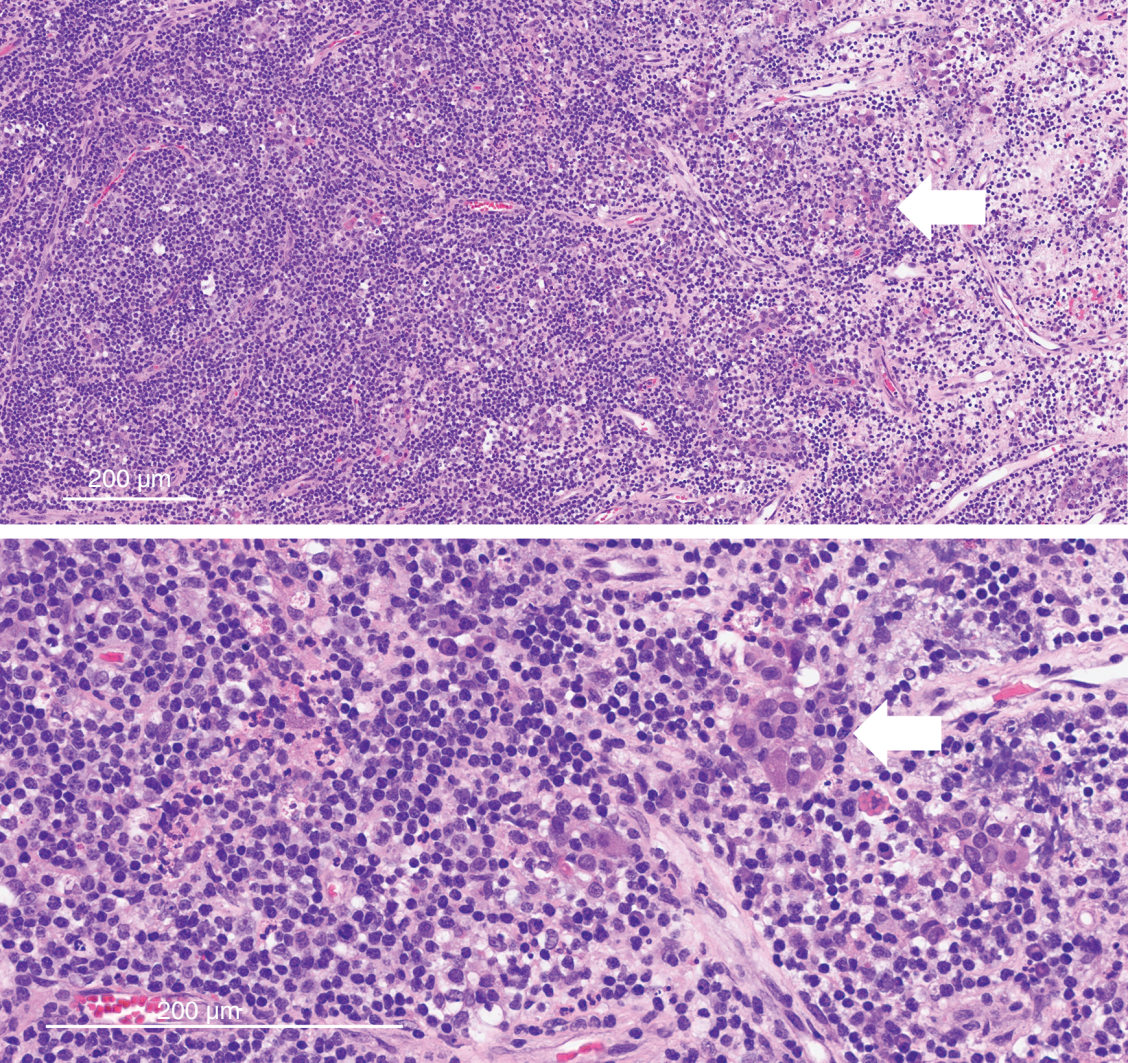
Lymphocytic hypophysitis—pathology. The adenohypophysis contains a dense inflammatory cell infiltrate that destroys the normal acinar architecture (top), with only a few residual adenohypophysial cells (arrow). On higher magnification (bottom), the inflammatory cells are mainly lymphocytes and plasma cells, and the adenohypophysial cells (arrow) have oncocytic cytoplasm.

In most instances assessment and monitoring of anterior and posterior pituitary function is all that is required with hormone replacement as required. Such hormone replacement needs to be re-assessed periodically to avoid long-term therapy that may not be indicated. The use of dopamine agonists to reduce the clinical consequences of associated hyperprolactinemia from a stalk effect is also of limited proven value.^46^ The use of corticosteroids to expedite the resolution of the inflammatory response to alter the course of the illness has been suggested. However, again, given the limited amount and quality of the data, this practice should be rigorously balanced against the potential negative consequences. In the more unusual instances where pituitary and surrounding structures show radiographic progression, neurosurgically guided tissue biopsy should be considered.^47^ In such instances, histopathologic examination (Figure 2) provides the opportunity to confirm the characteristic changes confirming the diagnosis. Furthermore, such tissue biopsy will serve to exclude other diagnoses as discussed below.

### Granulomatous Hypophysitis

#### Clinical setting.—

While this can occur as a primary and independent process that remains idiopathic, the more typical scenario is in the context of known or recently diagnosed systemic granulomatous disease such as infectious etiologies (tuberculous, fungal, or syphilitic), sarcoidosis or Wegener granulomatosis.^48–51^

Patients often present with headaches and an expanding sellar mass. Discrete involvement separate from the rest of the gland is often difficult to delineate providing a good clue to the diagnosis of this widely infiltrative disorder.

They can present as acute/subacute hypophysitis, either isolated or possibly more frequently in patients with HIV and other immunocompromised situations.^52^

#### Description and diagnosis.—

**Figure 3.**
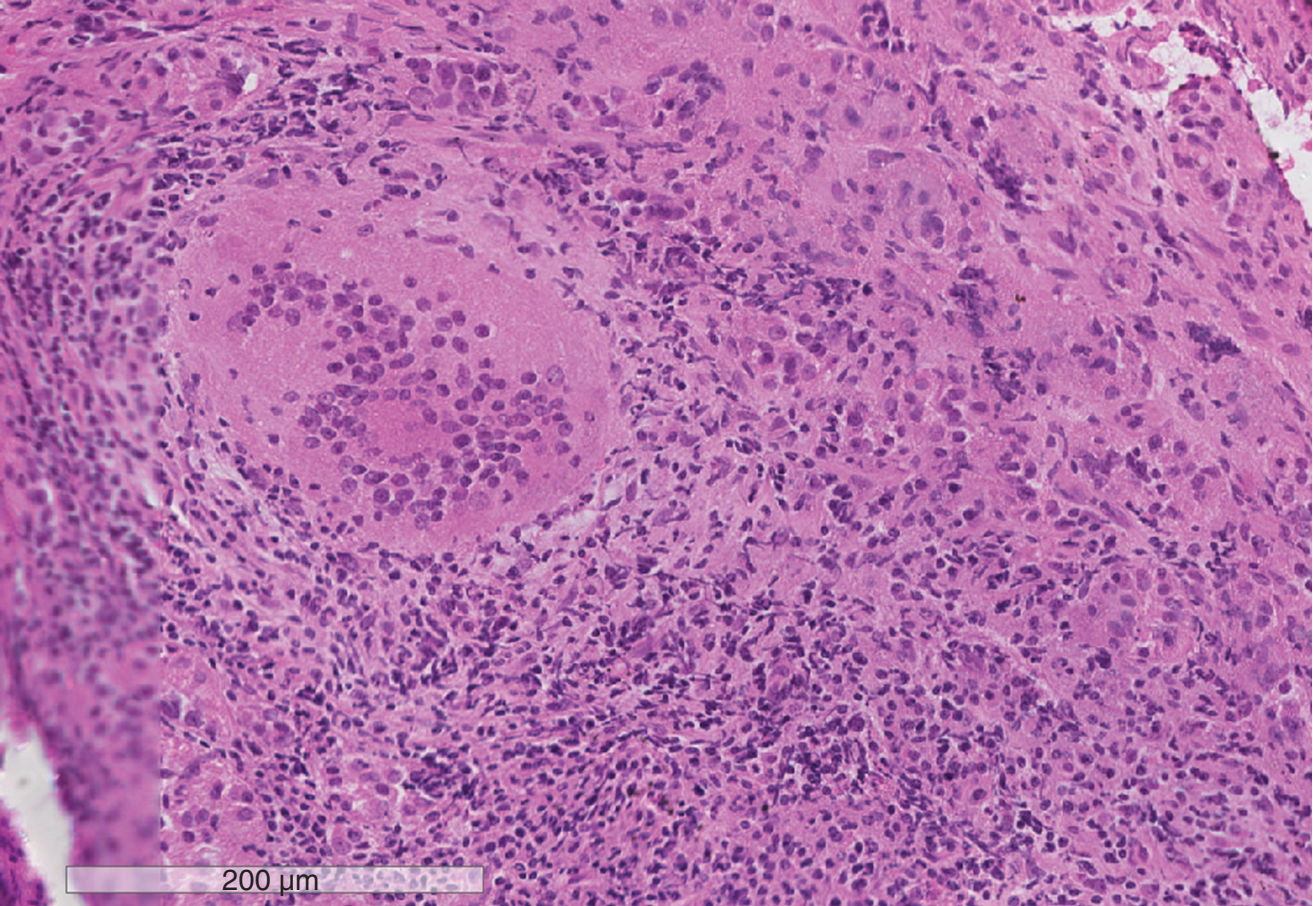
Granulomatous hypophysitis—pathology. The pituitary is infiltrated by a chronic inflammatory cell infiltrate with a large multinucleated giant cell.

Again, recognizing the potential involvement of the pituitary in patients with known granulomatous disease is usually straightforward. The more difficult cases are those where extra-pituitary disease is unknown. Serologic markers such as angiotensin-converting enzyme may provide a hint to the diagnosis of sarcoidosis. However, in its absence, diffuse enlargement of the pituitary provides the basis for an active search outside of the gland. Detection of lymph node enlargement either on clinical examination of the neck or radiographic imaging of the mediastinum provides supportive evidence.^53^ This also identifies potential sites for tissue biopsy to help establish the diagnosis (Figure 3). In general, access to lymph nodes in the neck or mediastinum provides a relatively less invasive opportunity for tissue examination avoiding the need for invasive pituitary surgery.

### Xanthomatous Hypophysitis

#### Clinical setting.—

While initially considered to be a rare primary pituitary disorder with characteristic infiltration by lipid-rich histiocytes it is now more frequently noted accompanying ruptured Rathke’s cleft cysts.^16^ As such, prior history or concomitant knowledge of a nearby Rathke’s cyst is important in considering this diagnosis. A related entity is seller xanthogranuloma^17^ that is usually associated with craniopharyngioma, but the dominant pathology in that entity is the infiltrative neoplasm.

#### Description and diagnosis.—

**Figure 4.**
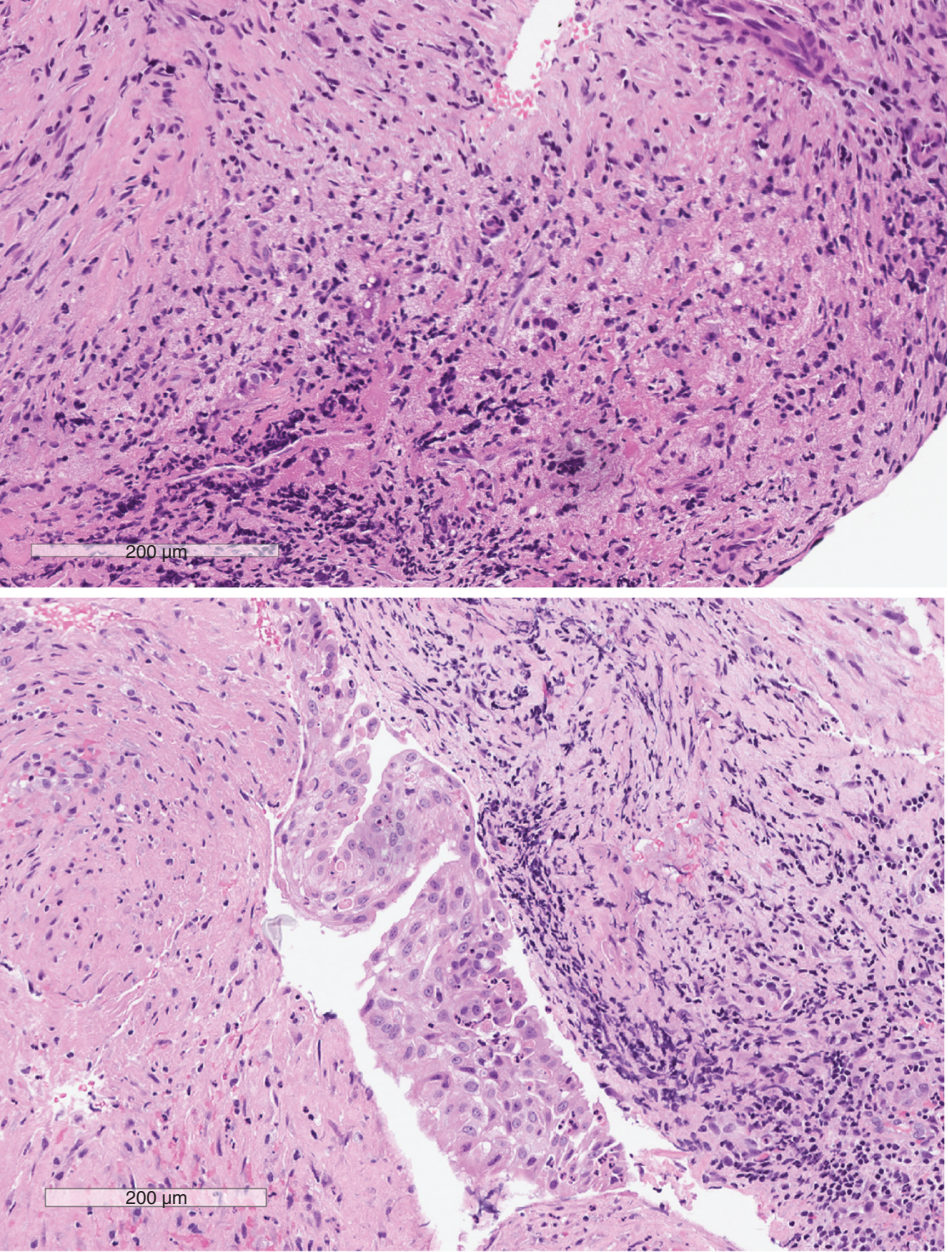
Xanthomatous hypophysitis associated with Rathke’s cleft cyst—pathology. The pituitary is distorted by an inflammatory cell infiltrate with numerous large histiocytes that have pale granular cytoplasm (top). Careful inspection identifies an associated fragment of the lining of a Rathke’s cleft cyst (bottom center) indicating that this inflammatory reaction is due to the rupture of the cyst and its associated keratin debris.

Histomorphologic examination is required for diagnosis (Figure 4) and there are no known circulating biomarkers. The identification of this disorder should prompt a careful examination of the imaging features to identify a cyst on MR imaging.

#### Therapeutic options.—

The use of anti-inflammatory agents has not been specifically examined or proven in this form of hypophysitis. As such, surgical decompression when indicated represents the mainstay of management. Unlike other forms of hypophysitis, recurrent cases are extremely rare.^54^

### IgG4 Hypophysitis

#### Clinical setting.—

**Figure 5.**
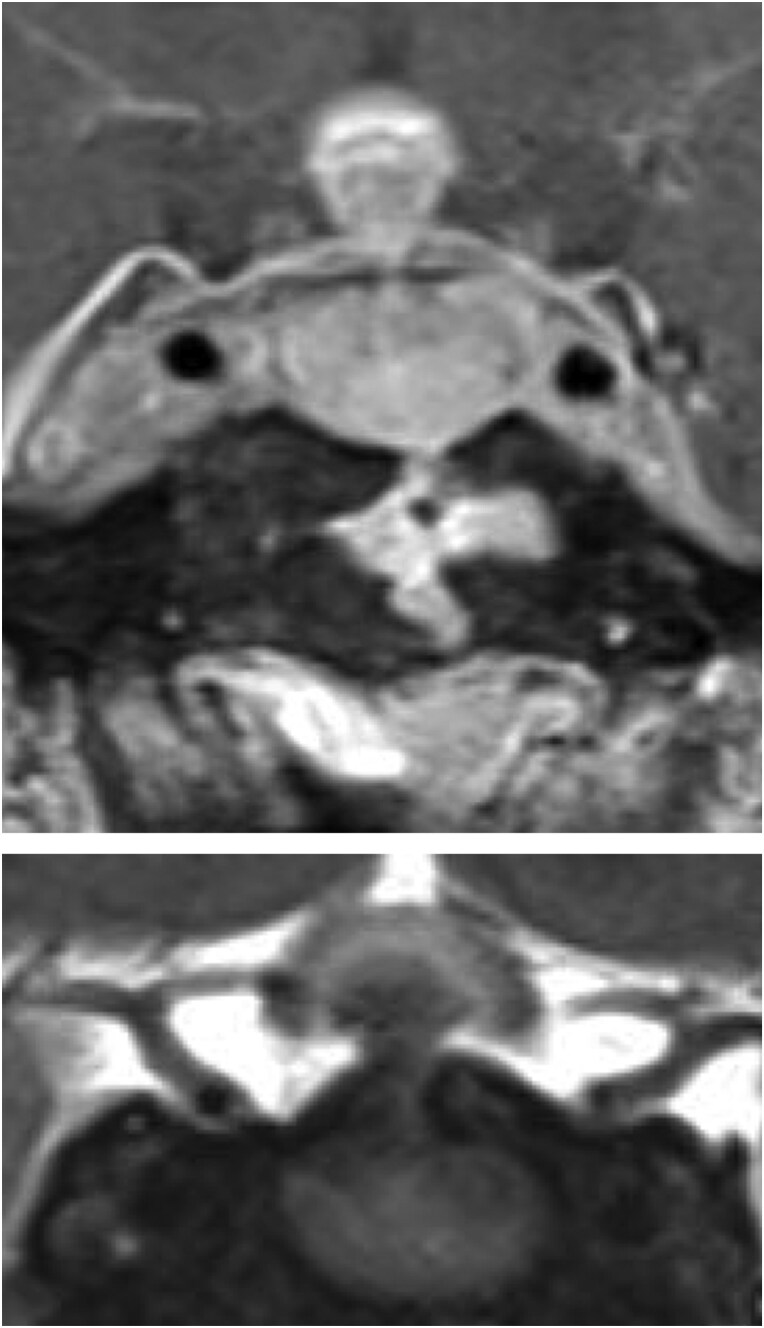
IgG4 hypophysitis—imaging. Left, postcontrast T1 pituitary magnetic resonance imaging coronal. There is infiltration of the pituitary gland, the infundibulum, and bilateral cavernous sinuses. The pituitary stalk is thickened with mass effect and upward bowing of the optic chiasm. Right, Coronal T2 weighted MRI shows associated mass effect and compression of the optic chiasm.

This immune-mediated plasma cell disorder results in infiltration of gastrointestinal, and head and neck organs with a highly fibrotic inflammatory process that causes tissue destruction and angiitis.^55^ Involvement of the pituitary, previously known as “inflammatory pseudotumor,” can follow or precede other organ infiltration (Figure 5). Heightened suspicion of pituitary disease should naturally occur in those with existing manifestations of IgG4 disease.^10,56^

#### Description and diagnosis.—

The diagnosis rests on tissue detection of IgG4 in the corresponding clinical setting. An elevated serum IgG4 level, typically above 100 mg/dl, is useful in supporting the diagnosis. However, the degree of elevation of IgG4 levels can sometimes be somewhat perplexing.^57^ Indeed, some studies have identified that up to 60% of patients with tissue-proven IgG4-related disease have normal serum levels of immunoglobulin. A falsely low or inappropriately normal IgG4 level can represent a laboratory artifact due to the so-called “hook” or prozone effect.^58^ This potential pitfall can be investigated by performing the assay with increased serum dilutions. Other immunoglobulin subclasses including IgG1, IgG2, and IgG3 can also be increased but to a lesser degree than IgG4. Serum complement levels are reduced consistent with enhanced consumption.^57^

#### Therapeutic options.—

Early diagnosis is imperative to improve overall outcomes and reduce the risk of relapse. Induction with high-dose glucocorticoids should be considered as remissions may not be durable with steroid tapering. Disease-modifying anti-rheumatic medications including azathioprine, mycophenolate, methotrexate, and cyclosporins have also been suggested.^59^ More recently, biologics such as the anti-CD20 rituximab have gained acceptance followed by anti-CTLA4 antibodies.^56^

### Infectious Hypophysitis

In addition to the infections mentioned above as causes of granulomatous hypophysitis, acute inflammation and the development of an abscess is a rare causes of a sellar mass. Prior history of meningitis or head and neck infection should prompt consideration of a pituitary abscess.^60^

SARS-CoV-2/COVID-19 has been associated with pituitary enlargement. Expression of angiotensin-converting enzyme 2 receptor has been implicated as the potential cause of involvement of the pituitary in those infected with the COVID-19 SARS-CoV-2 virus. Most case reports, however, have described such clinically relevant occurrences in the setting of preexisting macrotumors of the pituitary.^22^ It remains to be seen whether the virus alone can result in a tissue response mediating pituitary enlargement without or with failure of the gland.

### Immunotherapy Induced Hypophysitis

#### Clinical setting.—

Cancer immunotherapy is rapidly becoming the main clinical setting where pituitary dysfunction with or without radiographic enlargement is noted. There are probably many underlying reasons underpinning this phenomenon, the main one being the exquisite sensitivity of this gland to immune attack. In this regard, the use of anti-CTLA4 therapy with Ipilimumab ushered our recognition of immunotherapy-related hypophysitis.^61^

#### Description and diagnosis.—

The now well-recognized relationship between Ipilimumab and risk of hypophysitis in up to 17% of treated patients,^61^ has greatly facilitated awareness of this condition. Nevertheless, there continue to be many unclear concerns including the course of this complication, optimal therapy, and the risk of recurrence. What is clearer, however, is that such a risk of pituitary enlargement is considerably lower in patients receiving anti-PD or PDL-1 therapies than in those receiving anti-CTLA4 therapy.^62^ Regardless of immune target, the potential confounding effect of other therapies such as dexamethasone in patients receiving anti-PD-L1 pembrolizumab needs to be considered. While corticosteroids are often used to ameliorate adverse effects such as inflammatory and gastrointestinal effects, they may also contribute to cumulative effects on hypothalamic adrenal suppression.:

#### Therapeutic options.—

Usually, patients with immunotherapy-induced hypophysitis will only require hormone replacement.^12^ In the rare cases of severe mechanical symptoms (such as visual disturbances or intense headaches) glucocorticoids and/or decompression surgery may be needed.

Exclusion of metastasis in oncologic patients with sellar/suprasellar lesions is mandatory.

## Infiltrative Conditions

### Amyloidosis

The pituitary is a potential site of amyloid deposition.^63^ While this has been considered to be a feature noted on autopsy findings of elderly subjects,^64^ the extent to which it contributes to clinical manifestations remains relatively unknown.^65^ Additionally, amyloid deposits have been described in patients with preexisting pituitary tumors, further confounding its clinical significance.^66^

### Hemochromatosis

While iron deposition can occur throughout the endocrine system the anterior pituitary represents one potentially important site. This can occur with characteristic low signal on MRI.^67,68^ Impairment of pituitary function is not a consistent feature and can lag behind the imaging findings.^64^ Furthermore, the pattern of anterior hormone loss can be patchy, and unlike tumorous causes, can involve the gonadotroph or corticotroph axis despite normal somatotrophic function.^69–71^

## Conclusions and Recommendations

This review highlights the importance of careful clinicopathologic correlation when assessing enlargement of the pituitary. In many instances, a less-than-aggressive approach allows for a clearer picture to emerge. In situations where close surveillance notes evidence of structural disease progression, then neurosurgically driven biopsy and/or debulking approaches maybe warranted. Equally as important is the role of multidisciplinary tumor conferences where regular review of the pre and post-operative findings can ensure optimal management while enhancing quality care initiatives.
